# Supplementary material for: Overexpression of FRA1 (FOSL1) Leads to Global Transcriptional Perturbations, Reduced Cellular Adhesion and Altered Cell Cycle Progression
Source: Cells. 2023 Sep 24;12(19):2344. doi: 10.3390/cells12192344 (PMC10571788; doi:10.3390/cells12192344)
Supplement: Supplementary file 1 [file cells-12-02344-s001.zip › cells-2592798-supplementary.pdf]

Supplementary Materials

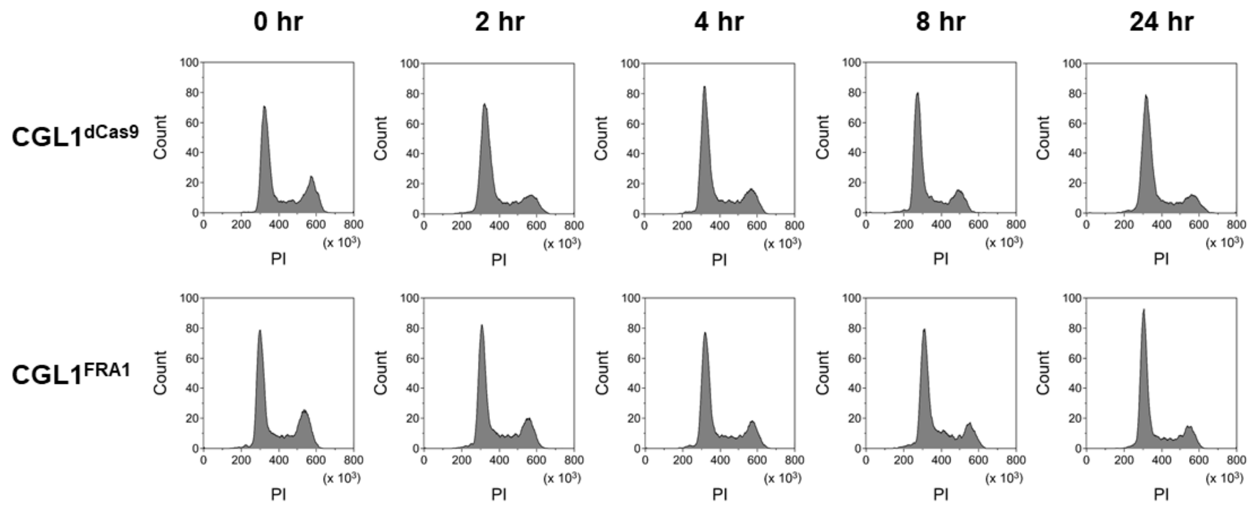

Supplementary Figure S1. Representative cell cycle distribution images of CGL1^{FRA1} and CGL1^{dCas9} cells. Cells were grown for 48h followed by complete media change. Samples were collected at the following time-points post media change: 2, 4, 8 and 24 hrs. Cell cycle phases were determined using propidium iodide (PI) nucleic acid fluorescent stain analyzed using a flow cytometer. Representative cell cycle distribution images of CGL1^{FRA1} and CGL1^{dCas9} cells are presented.

Supplementary Table S1. Full list of 298 DEGs in CGL1^{FRA1} cells relative to CGL1^{dCas9} cells presented in alphabetical order. Genes with fold change <-1.5 or >1.5, and FDR corrected p-value below 0.05 were assigned as DEGs. The table provides information on the fold change, p-value and TPM (transcript per million). The table contains 124 upregulated genes and 174 downregulated genes.

GENE ID	GENE NAME	FOLD CHANGE	P VALUE	CGL1^{dCas9} TPM	CGL1^{FRA1} TPM
ENSG00000268895.6	A1BG-AS1	-2.50	4.76E-02	26.1	10.5
ENSG00000108846.16	ABCC3	-2.11	1.13E-04	227.9	107.8
ENSG00000151388.11	ADAMTS12	1.75	2.14E-02	83.8	146.8
ENSG00000049192.15	ADAMTS6	2.44	9.20E-08	134.3	327.9
ENSG00000141385.9	AFG3L2	4.20	4.94E-08	38.2	160.3
ENSG00000155085.15	AK9	-1.73	4.28E-02	63.1	36.5
ENSG00000180318.4	ALX1	-1.65	4.94E-02	36.3	22.0
ENSG00000139211.6	AMIGO2	1.77	0.00E+00	147.0	260.5
ENSG00000165887.11	ANKRD2	-3.13	4.99E-02	47.3	15.1
ENSG00000230006.7	ANKRD36BP2	1.56	2.72E-02	77.6	121.4
ENSG00000138356.14	AOX1	1.87	5.55E-04	156.4	292.1
ENSG00000138613.14	APH1B	1.70	1.02E-02	93.4	158.9
ENSG00000128383.13	APOBEC3A	1.67	3.72E-02	28.6	47.7
ENSG00000221963.6	APOL6	-1.52	1.55E-02	431.3	284.4
ENSG00000178878.12	APOLD1	1.68	8.55E-06	222.9	375.5
ENSG00000047365.12	ARAP2	-3.32	1.32E-02	107.0	32.2
ENSG00000004777.18	ARHGAP33	2.99	3.42E-02	28.2	84.2
ENSG00000116017.11	ARID3A	1.72	2.46E-02	157.4	270.1
ENSG00000188042.8	ARL4C	-1.85	1.41E-05	167.2	90.5
ENSG00000006756.16	ARSD	-1.67	3.64E-02	110.9	66.3
ENSG00000116539.13	ASH1L	1.53	1.40E-02	415.1	634.1
ENSG00000110881.11	ASIC1	-3.37	4.26E-03	103.7	30.7
ENSG00000271614.1	ATP2B1-AS1	-2.25	3.17E-02	23.5	10.4
ENSG00000033627.16	ATP6V0A1	1.61	1.44E-04	822.0	1322.2
ENSG00000254870.5	ATP6V1G2-DDX39B	1.68	4.96E-02	67.7	113.6
ENSG00000162650.16	ATXN7L2	1.61	1.60E-02	62.1	99.9
ENSG00000148090.11	AUH	-1.90	3.67E-02	31.2	16.5
ENSG00000127423.10	AUNIP	162.21	1.17E-07	0.3	52.2
ENSG00000168646.13	AXIN2	-1.88	4.02E-02	290.3	154.3
ENSG00000087088.20	BAX	2.10	3.76E-02	2678.8	5551.6
ENSG00000138756.17	BMP2K	-1.72	1.05E-02	113.5	65.9
ENSG00000168014.16	C2CD3	-1.53	4.19E-02	77.7	50.8
ENSG00000115998.7	C2orf42	4.44	1.15E-02	5.9	26.2
ENSG00000187699.10	C2orf88	1.89	1.38E-02	25.2	47.7
ENSG00000088543.15	C3orf18	-1.60	1.36E-02	79.8	49.8

ENSG00000224389.9	C4B	-2.55	1.78E-02	23.4	9.2
ENSG00000147894.16	C9orf72	-1.59	4.00E-02	65.6	41.3
ENSG00000177640.16	CASC2	-2.56	2.03E-02	23.4	9.1
ENSG00000239521.8	CASTOR3	-1.75	3.05E-02	159.1	90.9
ENSG00000183287.14	CCBE1	1.83	3.47E-03	59.5	108.7
ENSG00000135205.15	CCDC146	2.65	2.70E-02	11.2	29.7
ENSG00000197816.14	CCDC180	2.16	1.30E-02	35.8	77.4
ENSG00000149231.14	CCDC82	-1.60	2.85E-02	408.9	255.9
ENSG00000173013.6	CCDC96	-1.93	4.66E-02	27.0	14.0
ENSG00000108691.9	CCL2	-1.58	1.43E-02	133.4	84.4
ENSG00000271503.6	CCL5	-3.21	5.81E-03	54.1	16.9
ENSG00000204936.10	CD177	1.58	4.84E-02	32.8	51.6
ENSG00000272398.6	CD24	-1.70	3.97E-02	102.0	60.1
ENSG00000215039.7	CD27-AS1	1.95	2.08E-02	45.3	88.3
ENSG00000171219.9	CDC42BPG	3.52	1.54E-02	6.7	23.8
ENSG00000163171.7	CDC42EP3	1.52	4.44E-02	88.8	134.7
ENSG00000156345.17	CDK20	-1.55	3.28E-02	151.4	97.4
ENSG00000221869.5	CEBPD	-1.55	4.92E-03	371.3	239.6
ENSG00000188312.14	CENPP	1.56	2.93E-02	363.6	567.9
ENSG00000154645.14	CHODL	2.33	1.81E-02	11.4	26.6
ENSG00000125954.12	CHURC1-FNTB	-3.86	1.39E-02	43.5	11.3
ENSG00000159208.16	CIART	-1.72	4.09E-02	47.4	27.5
ENSG00000164309.15	CMYA5	-2.04	2.92E-02	33.0	16.2
ENSG00000187955.12	COL14A1	-2.76	1.24E-03	96.5	35.0
ENSG00000168542.14	COL3A1	1.53	1.56E-02	846.6	1292.9
ENSG00000158270.12	COLEC12	-2.51	1.54E-02	44.3	17.7
ENSG00000135469.13	COQ10A	1.52	1.49E-02	122.5	186.2
ENSG00000104324.16	CPQ	-1.53	8.49E-03	159.9	104.7
ENSG00000021826.16	CPS1	1.56	0.00E+00	25.8	40.1
ENSG00000198223.16	CSF2RA	2.02	6.93E-03	40.5	81.8
ENSG00000107562.16	CXCL12	1.55	3.74E-02	149.0	230.6
ENSG00000145476.16	CYP4V2	-1.55	2.44E-02	128.7	83.2
ENSG00000035664.11	DAPK2	5.90	5.73E-04	7.9	46.4
ENSG00000145041.15	DCAF1	1.86	3.27E-03	253.3	471.5
ENSG00000011465.17	DCN	-1.61	3.68E-03	1764.2	1097.2
ENSG00000134574.11	DDB2	1.68	3.40E-05	113.1	189.7
ENSG00000107201.10	DDX58	-2.62	2.83E-03	271.7	103.7
ENSG00000137628.17	DDX60	-1.98	1.61E-03	582.5	294.5
ENSG00000100150.19	DEPDC5	1.64	3.77E-03	129.5	213.0
ENSG00000099958.14	DERL3	-1.72	1.48E-02	103.8	60.4
ENSG00000162496.9	DHRS3	-3.29	1.28E-02	419.5	127.5
ENSG00000108771.13	DHX58	-2.05	2.88E-02	37.8	18.4

ENSG00000155011.9	DKK2	-2.20	3.62E-02	25.1	11.4
ENSG00000150768.15	DLAT	3.34	2.69E-02	16.5	55.1
ENSG00000125375.15	DMAC2L	-1.87	1.96E-02	109.7	58.7
ENSG00000104936.17	DMPK	1.69	2.01E-02	263.1	443.4
ENSG00000135392.16	DNAJC14	2.76	6.31E-03	53.4	147.7
ENSG00000197959.14	DNM3	-1.77	1.93E-03	93.1	52.6
ENSG00000173852.14	DPY19L1	-3.62	3.42E-02	48.5	13.4
ENSG00000115468.12	EFHD1	-1.90	1.08E-02	75.7	39.8
ENSG00000184349.13	EFNA5	-1.56	8.88E-03	381.6	243.8
ENSG00000138798.13	EGF	-2.71	4.91E-03	25.1	9.3
ENSG00000032389.12	EIPR1	1.60	4.19E-02	43.1	68.9
ENSG00000118985.16	ELL2	1.53	2.89E-04	2077.0	3182.4
ENSG00000120658.13	ENOX1	-2.75	2.89E-03	72.9	26.5
ENSG00000135476.11	ESPL1	1.62	9.26E-03	205.7	333.6
ENSG00000067208.14	EVI5	1.67	3.35E-04	183.9	307.2
ENSG00000121104.8	FAM117A	1.80	1.27E-02	36.6	65.9
ENSG00000196159.11	FAT4	-1.99	0.00E+00	20.2	10.1
ENSG00000113578.18	FGF1	1.88	9.62E-03	25.0	47.0
ENSG00000174721.10	FGFBP3	-1.86	2.01E-02	42.9	23.0
ENSG00000176971.4	FIBIN	-2.62	5.41E-06	96.8	36.9
ENSG00000155816.20	FMN2	1.54	1.23E-02	367.4	565.7
ENSG00000115414.19	FN1	-2.01	4.42E-03	8860.3	4769.6
ENSG00000170345.10	FOS	-1.53	1.65E-03	518.3	314.7
ENSG00000175592.9	FOSL1	2.04	1.86E-02	4086.2	6317.1
ENSG00000179772.8	FOXS1	-4.12	1.68E-03	34.0	8.3
ENSG00000073910.22	FRY	-5.00	0.00E+00	203.2	40.6
ENSG00000157240.4	FZD1	-1.74	4.57E-02	1102.7	633.0
ENSG00000109458.8	GAB1	-1.55	2.73E-02	224.2	144.8
ENSG00000141429.13	GALNT1	-1.74	3.65E-03	978.0	561.8
ENSG00000007237.18	GAS7	-7.15	2.76E-04	50.4	7.0
ENSG00000144649.9	GASK1A	3.18	1.27E-02	7.2	22.8
ENSG00000130513.6	GDF15	-2.18	0.00E+00	298.0	136.9
ENSG00000178445.9	GLDC	-3.30	4.51E-04	76.0	23.0
ENSG00000167699.13	GLOD4	1.53	1.77E-02	134.4	205.6
ENSG00000087258.15	GNAO1	-1.60	3.10E-02	76.4	47.9
ENSG00000127920.6	GNG11	-1.55	1.21E-03	217.5	139.9
ENSG00000138678.11	GPAT3	1.64	3.92E-02	36.5	59.6
ENSG00000170775.3	GPR37	1.73	2.16E-04	89.0	154.1
ENSG00000100077.15	GRK3	-3.80	2.65E-03	51.5	13.5
ENSG00000104518.11	GSDMD	-1.52	4.65E-02	190.6	125.3
ENSG00000103044.11	HAS3	2.13	8.63E-04	47.7	101.5
ENSG00000143575.14	HAX1	-1.92	4.02E-02	107.4	56.1
ENSG00000130589.16	HELZ2	-1.86	3.17E-02	80.2	43.1

ENSG00000138646.9	HERC5	-1.81	1.26E-02	174.1	96.1
ENSG00000069812.11	HES2	-2.33	4.32E-02	36.9	15.8
ENSG00000181218.5	HIST3H2A	-1.61	8.68E-04	205.7	127.4
ENSG00000156515.23	HK1	1.70	2.28E-02	108.7	185.4
ENSG00000171004.18	HS6ST2	-1.64	1.07E-02	376.8	229.6
ENSG00000086696.11	HSD17B2	-2.76	2.35E-02	112.7	40.9
ENSG00000137965.11	IFI44	-1.75	2.20E-04	960.7	548.7
ENSG00000115267.8	IFIH1	-1.82	1.21E-02	106.9	58.8
ENSG00000119917.14	IFIT3	-1.60	5.44E-03	1090.7	681.1
ENSG00000159128.14	IFNGR2	1.80	4.56E-03	50.9	91.6
ENSG00000141753.7	IGFBP4	-1.54	9.49E-03	150.9	98.2
ENSG00000163453.11	IGFBP7	-1.57	5.68E-03	986.1	626.2
ENSG00000134352.20	IL6ST	3.05	2.32E-06	46.5	141.9
ENSG00000163362.11	INAVA	-3.46	2.45E-02	26.4	7.6
ENSG00000274049.4	INO80B-WBP1	-5.81	1.53E-02	60.7	10.5
ENSG00000103599.20	IQCH	-2.33	3.56E-03	38.5	16.5
ENSG00000185507.21	IRF7	-1.58	1.38E-02	83.5	53.0
ENSG00000187608.10	ISG15	-1.73	9.49E-05	1935.1	1120.8
ENSG00000137809.17	ITGA11	-2.07	9.13E-09	1286.9	622.2
ENSG00000115232.14	ITGA4	2.71	4.92E-02	1041.6	3147.2
ENSG00000105855.10	ITGB8	-1.60	3.28E-02	922.4	616.4
ENSG00000150995.19	ITPR1	1.54	4.38E-02	191.3	294.3
ENSG00000198885.9	ITPRIPL1	-1.59	1.61E-02	91.0	57.2
ENSG00000168970.22	JMJD7-PLA2G4B	1.53	2.63E-03	121.6	186.2
ENSG00000107077.18	KDM4C	1.53	3.01E-02	102.9	157.9
ENSG00000066735.14	KIF26A	1.75	0.00E+00	37.4	65.3
ENSG00000174010.9	KLHL15	2.31E+06	0.00E+00	0.0	59.7
ENSG00000055609.18	KMT2C	-1.73	2.52E-02	459.7	266.5
ENSG00000128422.17	KRT17	-3.10	4.55E-02	348.1	112.3
ENSG00000170477.13	KRT4	-1.89	1.33E-02	411.7	217.4
ENSG00000186615.11	KTN1-AS1	1.97	1.21E-03	52.7	103.9
ENSG00000185513.16	L3MBTL1	1.75	1.28E-03	68.9	120.4
ENSG00000213626.13	LBH	-2.21	4.99E-02	505.7	229.2
ENSG00000135338.14	LCA5	-1.52	7.75E-03	113.8	75.0
ENSG00000138795.10	LEF1	-1.55	3.74E-02	64.1	41.3
ENSG00000121454.6	LHX4	-2.28	1.91E-02	62.3	27.4
ENSG00000238266.2	LINC00707	1.76	4.32E-02	75.1	132.2
ENSG00000175772.11	LINC01106	-1.65	3.16E-03	217.2	131.5
ENSG00000234840.2	LINC01239	-2.21	8.63E-03	49.9	22.5
ENSG00000248677.2	LINC02102	-5.24	3.76E-02	29.9	5.7
ENSG00000079435.10	LIPE	1.91	5.51E-04	60.5	115.9

ENSG00000184709.7	LRRC26	-1.97	2.28E-02	89.3	45.3
ENSG00000185158.12	LRRC37B	-1.68	4.19E-02	56.5	33.7
ENSG00000119681.12	LTBP2	-1.74	1.35E-02	440.1	252.5
ENSG00000183060.15	LYSMD4	-1.71	2.39E-02	100.4	58.7
ENSG00000107816.17	LZTS2	-1.54	1.59E-02	547.9	356.9
ENSG00000172264.17	MACROD2	-4.92	2.32E-06	39.8	8.1
ENSG00000102158.19	MAGT1	-3.58	4.42E-03	33.9	9.5
ENSG00000162959.13	MEMO1	-1.54	2.23E-02	309.1	200.8
ENSG00000117122.14	MFAP2	-2.29	2.52E-04	416.7	182.2
ENSG00000167889.12	MGAT5B	2.78	5.53E-04	46.2	128.5
ENSG00000105556.11	MIER2	-2.30	8.24E-03	45.6	19.9
ENSG00000262454.4	MIR193BHG	-1.72	1.67E-02	319.7	186.4
ENSG00000087245.13	MMP2	-1.58	3.27E-04	1088.7	690.4
ENSG00000149968.12	MMP3	-2.49	7.46E-05	91.1	36.6
ENSG00000101825.8	MXRA5	-1.72	2.19E-02	473.0	275.8
ENSG00000118513.19	MYB	-2.11	7.75E-04	132.4	62.6
ENSG00000065534.18	MYLK	-2.21	1.43E-02	274.0	123.8
ENSG00000244754.8	N4BP2L2	1.80	2.03E-02	82.0	147.7
ENSG00000188613.7	NANOS1	-1.79	2.91E-02	62.1	34.7
ENSG00000132688.11	NES	-1.73	2.54E-02	87.6	50.5
ENSG00000101096.20	NFATC2	-1.93	2.06E-02	46.2	23.9
ENSG00000100906.10	NFKBIA	-2.54	1.58E-02	64.9	25.5
ENSG00000170322.14	NFRKB	1.62	7.61E-03	444.6	720.6
ENSG00000169760.17	NLGN1	3.51	9.37E-09	53.1	186.3
ENSG00000165246.14	NLGN4Y	-2.15	1.25E-02	67.2	31.3
ENSG00000175352.11	NRIP3	1.59	4.53E-02	44.9	71.5
ENSG00000142619.4	PADI3	-2.32	2.01E-02	25.8	11.1
ENSG00000227345.8	PARG	1.58	4.14E-02	124.2	195.8
ENSG00000276547.1	PCDHGB5	-81.82	4.48E-02	54.0	0.7
ENSG00000100311.17	PDGFB	-1.90	1.38E-02	215.4	113.4
ENSG00000164530.15	PI16	-1.80	3.61E-02	173.1	96.0
ENSG00000165282.14	PIGO	4.06	1.95E-11	78.6	318.9
ENSG00000100100.13	PIK3IP1	-1.94	8.16E-03	114.0	58.8
ENSG00000133321.11	PLAAT4	-2.99	3.36E-04	129.4	43.3
ENSG00000116095.11	PLEKHA3	1.76	4.51E-04	263.6	465.3
ENSG00000198753.12	PLXNB3	3.98	1.54E-04	14.0	55.9
ENSG00000101868.11	POLA1	-2.69	4.35E-02	491.0	182.3
ENSG00000175482.9	POLD4	-1.57	1.59E-03	317.6	201.9
ENSG00000228049.7	POLR2J2	2.22	3.03E-02	19.0	42.1
ENSG00000102103.16	PQBP1	1.55	3.05E-02	104.2	161.9
ENSG00000184500.15	PROS1	7.57	1.59E-02	5.3	40.4
ENSG00000185920.15	PTCH1	-1.53	4.73E-03	563.9	369.0
ENSG00000165983.14	PTER	1.77	1.88E-03	135.4	239.8

ENSG00000088179.9	PTPN4	1.71	2.80E-03	412.2	706.2
ENSG00000255857.6	PXN-AS1	4.41	6.87E-04	11.5	50.8
ENSG00000076864.19	RAP-1GAP	-1.63	3.94E-02	137.1	84.3
ENSG00000109756.9	RAPGEF2	1.52	8.91E-03	476.4	725.5
ENSG00000265666.1	RARA-AS1	-2.31	3.99E-02	27.1	11.7
ENSG00000242875.6	RBMY1B	-1.52	4.92E-03	170.6	112.3
ENSG00000226941.8	RBMY1J	2.74	0.00E+00	84.9	232.3
ENSG00000143344.15	RGL1	-2.22	4.38E-03	97.1	43.7
ENSG00000204130.13	RUFY2	-2.80	3.58E-05	71.0	25.3
ENSG00000228956.8	SATB1-AS1	-2.17	3.30E-02	23.3	10.7
ENSG00000164764.11	SBSPON	-1.77	3.45E-02	66.3	37.4
ENSG00000171222.10	SCAND1	-1.69	1.98E-03	158.8	93.9
ENSG00000006747.15	SCIN	2.71	2.66E-03	10.5	28.5
ENSG00000260428.3	SCX	-1.71	4.01E-02	75.5	44.0
ENSG00000205572.9	SERF1B	-4.70	6.11E-04	130.0	27.7
ENSG00000135919.13	SERPINE2	-1.73	1.85E-04	535.8	308.9
ENSG00000136169.16	SETDB2	5.52	1.39E-02	6.3	34.7
ENSG00000145423.5	SFRP2	-2.44	2.31E-02	65.8	26.9
ENSG00000169247.12	SH3TC2	-1.84	3.78E-02	109.8	59.8
ENSG00000168779.19	SHOX2	-2.18	7.82E-05	217.8	99.8
ENSG00000124523.16	SIRT5	-1.61	6.84E-03	147.9	91.8
ENSG00000197375.12	SLC22A5	2.76	2.27E-02	21.2	58.5
ENSG00000181625.17	SLX1B	-1.98	4.08E-02	137.9	69.7
ENSG00000072501.17	SMC1A	1.58	1.10E-02	211.3	334.4
ENSG00000095637.22	SORBS1	1.68	4.26E-02	32.5	54.6
ENSG00000124766.7	SOX4	-1.59	4.41E-02	3527.7	2213.8
ENSG00000105866.15	SP4	-1.53	6.33E-06	549.2	358.1
ENSG00000104450.12	SPAG1	12.20	0.00E+00	2.8	33.8
ENSG00000166145.14	SPINT1	3.31	4.75E-02	7.5	24.7
ENSG00000196935.9	SRGAP-1	1.86	4.79E-03	242.4	451.3
ENSG00000196369.11	SRGAP2B	5.47	5.94E-03	58.2	318.6
ENSG00000139874.6	SSTR1	1.80	1.00E-03	379.6	681.9
ENSG00000125046.15	SSUH2	1.95	4.44E-03	37.1	72.3
ENSG00000123473.15	STIL	2.49	1.62E-05	61.4	152.9
ENSG00000213533.12	STIMATE	2.71	4.61E-02	8.2	22.3
ENSG00000137868.19	STRA6	-3.34	2.62E-03	553.8	165.9
ENSG00000166263.13	STXBP4	1.84	1.21E-03	250.1	460.6
ENSG00000196562.14	SULF2	-3.71	5.99E-06	222.5	60.0
ENSG00000204176.14	SYT15	3.63	3.76E-03	8.8	32.1
ENSG00000152284.5	TCF7L1	-2.48	2.96E-02	44.8	18.1
ENSG00000167074.15	TEF	-1.72	2.12E-02	147.5	85.9
ENSG00000120708.17	TGFBI	-1.69	1.93E-02	62196.7	36860.6
ENSG00000198959.12	TGM2	-2.03	4.35E-02	39.9	19.6

ENSG00000151090.19	THRB	1.81	1.07E-02	59.1	107.2
ENSG00000149809.15	TM7SF2	1.79	3.49E-02	34.5	61.7
ENSG00000184988.8	TMEM106A	1.52	2.99E-02	53.4	81.1
ENSG00000170006.12	TMEM154	1.99	4.22E-02	100.0	198.5
ENSG00000187824.8	TMEM220	2.66	4.89E-02	8.8	23.4
ENSG00000262481.5	TMEM256-PLSCR3	2.80	3.49E-02	9.1	25.4
ENSG00000231770.6	TMEM44-AS1	2.56	3.93E-02	19.6	50.1
ENSG00000254860.6	TMEM9B-AS1	-4.58	1.32E-04	64.3	14.1
ENSG00000158164.7	TMSB15A	-1.96	4.06E-03	90.7	46.2
ENSG00000161955.16	TNFSF13	1.75	1.39E-02	39.1	68.5
ENSG00000154310.17	TNIK	1.70	9.49E-04	154.5	262.8
ENSG00000167632.16	TRAPPC9	-2.02	2.23E-03	243.4	120.4
ENSG00000255690.3	TRIL	-2.42	1.19E-02	66.9	27.7
ENSG00000204977.10	TRIM13	-1.81	5.95E-05	249.3	137.9
ENSG00000248275.2	TRIM52-AS1	-1.63	4.16E-02	65.0	39.9
ENSG00000130529.16	TRPM4	1.68	4.37E-03	70.0	117.3
ENSG00000182463.16	TSHZ2	1.54	3.27E-02	93.6	144.3
ENSG00000259024.6	TVP23C-CDRT4	1.53	9.60E-03	126.1	193.4
ENSG00000120942.13	UBIAD1	-2.04	3.56E-02	51.1	25.1
ENSG00000076248.10	UNG	-2.40	3.10E-07	313.8	130.8
ENSG00000106608.16	URGCP	2.22	1.04E-02	124.1	275.2
ENSG00000138768.14	USO1	2.99	4.60E-02	20.6	61.7
ENSG00000253797.2	UTP14C	-6.31E+06	0.00E+00	31.9	0.0
ENSG00000038427.16	VCAN	-2.66	3.24E-03	101.9	38.3
ENSG00000147852.16	VLDLR	-2.08	2.27E-02	42.7	20.5
ENSG00000158023.10	WDR66	2.09	2.48E-02	17.4	36.4
ENSG00000261040.7	WFDC21P	4.94	2.37E-03	12.1	60.0
ENSG00000143816.8	WNT9A	-1.87	2.64E-02	164.5	87.9
ENSG00000234825.3	XRCC6P2	-1.55	2.73E-02	65.3	42.2
ENSG00000247271.7	ZBED5-AS1	2.19	3.27E-02	16.2	35.5
ENSG00000168826.16	ZBTB49	1.79	4.68E-02	47.7	85.3
ENSG00000184307.15	ZDHHC23	-2.08	4.66E-02	111.8	53.9
ENSG00000147118.11	ZNF182	-2.91	5.68E-04	127.8	44.0
ENSG00000122386.10	ZNF205	-1.88	1.50E-02	157.9	83.8
ENSG00000159885.14	ZNF222	-1.56	1.76E-02	85.7	55.0
ENSG00000167840.13	ZNF232	-1.75	4.47E-02	81.6	46.7
ENSG00000256771.4	ZNF253	1.53	4.12E-02	47.3	72.3
ENSG00000180855.16	ZNF443	-1.69	2.75E-02	59.4	35.1
ENSG00000173258.13	ZNF483	2.10	2.78E-02	27.5	57.9
ENSG00000144026.12	ZNF514	1.58	3.35E-02	146.1	231.5
ENSG00000187187.14	ZNF546	-1.53	1.75E-02	74.7	48.8

ENSG00000245680.10	ZNF585B	-1.62	2.93E-02	62.9	38.7
ENSG00000166704.11	ZNF606	-2.34	8.38E-03	69.7	29.8
ENSG00000197928.10	ZNF677	1.57	3.44E-02	34.9	54.9
ENSG00000237440.9	ZNF737	-6.62	2.27E-03	31.5	4.8
ENSG00000160336.15	ZNF761	7.86	4.85E-03	4.0	31.3
ENSG00000198482.13	ZNF808	-1.98	9.38E-03	87.2	44.0
ENSG00000167766.18	ZNF83	1.96	2.04E-04	115.6	226.4
ENSG00000197608.11	ZNF841	2.46	1.78E-06	58.0	142.4
ENSG00000140265.12	ZSCAN29	4.26	5.23E-10	56.2	239.8
ENSG00000235109.7	ZSCAN31	-1.64	4.38E-02	50.6	30.8
